# Die primäre und die psychiatrisch-„komorbide“, nichtorganische Insomnie in einem neurologisch geführten Schlaflabor

**DOI:** 10.1007/s00739-022-00790-z

**Published:** 2022-03-01

**Authors:** Sarah Deutsch-Lang, Maria Kuchling, Isabel Valeske, Petra Hulle-Wegl, Robert Stepansky, Wilfried Lang

**Affiliations:** 1 Fakultät für Psychotherapiewissenschaften, Sigmund Freud Privatuniversität, Freudplatz 1, 1020 Wien, Österreich; 2 Fakultät für Medizin, Sigmund Freud Privatuniversität, Wien, Österreich; 3 Abteilung für Neurologie, neurologische Rehabilitation und Akutgeriatrie, Krankenhaus der Barmherzigen Brüder Wien, Johannes von Gott Platz 1, 1020 Wien, Österreich

**Keywords:** Primäre Insomnie, Sekundäre Insomnie, Psychiatrische Komorbidität bei Insomnie, Kognitive Verhaltenstherapie der Insomnie, Schlaflabor, Primary insomnia, Secondary insomnia, Psychiatric comorbidity in insomnia, Cognitive behavioral therapy for insomnia, Sleep laboratory

## Abstract

Patientinnen und Patienten, die in einem neurologischen Schlaflabor die Abschlussdiagnose nichtorganische Insomnie erhalten, leiden zu einem großen Teil (34 von 43 Personen) unter einer psychiatrischen Erkrankung: Persönlichkeitsstörungen, mit und ohne zusätzliche affektive Störung, Depression, Dysthymie, Zyklothymie, bipolare Störung, Angststörungen (generalisierte Angststörung, soziale Phobie), somatoforme (autonome) Funktionsstörung, hypochondrische Störung, Zwangsstörung, Anpassungsstörungen nach dramatischen Lebensereignissen sowie posttraumatische Belastungsstörungen. Sie befinden sich in laufender psychiatrischer und psychotherapeutischer Behandlung, leiden aber anhaltend unter den Symptomen der Insomnie. Allen Patientinnen und Patienten mit nichtorganischer Insomnie wird die kognitive Verhaltenstherapie der Insomnie angeboten.

## Abstract (en)

Patients who receive the diagnosis of non-organic insomnia in a neurological sleep laboratory mostly suffer from psychiatric disorders (34 out of 43 persons): personality disorders, with or without additional affective disorder, depression, dysthymia, cyclothymia, bipolar disorder, anxiety (generalized anxiety disorder, social phobia), somatoform (autonomous) disorder, hypochondriacal disorder, obsessive-compulsive disorder, adjustment disorders after dramatic life events, and post-traumatic stress disorder. They are under permanent psychiatric treatment and psychotherapy, but they still suffer from symptoms of insomnia. All patients with non-organic insomnia are offered cognitive behavioral therapy for insomnia.

## Einleitung

Die Diagnose nichtorganische Insomnie (F51.0) wird in der Regel durch Anamnese, spezielle Fragebögen und Schlaftagebücher gestellt. Grundsätzlich besteht eine Indikation zur Polysomnographie im Schlaflabor bei Vorliegen einer schweren Insomnie mit signifikanter Beeinträchtigung der Tagesbefindlichkeit oder Vorliegen einer „therapieresistenten“ Insomnie über mehr als ein halbes Jahr [1]. In einem 3‑Monats-Zeitraum (01.10.2018–31.01.2019) erhielten im Schlaflabor des Krankenhauses der Barmherzigen Brüder Wien 76 von 291 Personen die Diagnose nichtorganische Insomnie (F51.0), entweder alleine (50 von 291, ca. 17 %) oder in Kombination mit anderen Schlafstörungen (atmungsbezogene Schlafstörung bzw. therapierelevantes Restless-Legs-Syndrom; [2]). Beginnend mit Oktober 2021 wurden konsekutiv alle Patientinnen und Patienten, die im Schlaflabor des Krankenhauses der Barmherzigen Brüder die Diagnose nichtorganische Insomnie erhalten hatten, eingeladen, an strukturierten Interviews und psychodiagnostischen Untersuchungen teilzunehmen. Ziel des Projekts ist, das Spektrum der bestehenden psychiatrischen Komorbidität zu beschreiben, die Personen zu einem Therapieprogramm (kognitive Verhaltenstherapie der Insomnie) einzuladen und den Erfolg der Therapie zu evaluieren. Wir berichten hier einerseits über das Konzept unseres Projekts („Interdisziplinäre Kooperation zur Behandlung der Insomnie“, Kooperation Barmherzige Brüder Wien – Sigmund Freud Privatuniversität) und andererseits über Häufigkeit und Spektrum der psychiatrischen Komorbidität.

## Definition der nichtorganischen Insomnie

Die (persistierende), nichtorganische Insomnie (F 51.0) wird aufgrund der Dauer der Beschwerden von mehr als drei Monaten von der akuten (episodischen), „anpassungsbedingten“ Insomnie abgegrenzt. Beklagt werden Einschlafstörungen, Durchschlafstörungen mit häufigen und verlängerten Wachphasen sowie ein vorzeitiges Erwachen. Die insomnischen Beschwerden sind häufig (≥ 3 Nächte/Woche) und beeinträchtigen Tagesbefinden und Lebensqualität. Hier im Vordergrund stehen: Müdigkeit, Beeinträchtigungen von Aufmerksamkeit, Konzentration, Gedächtnis oder Stimmung (Gereiztheit, Irritabilität), soziale und berufliche Einschränkungen, erhöhte Fehleranfälligkeit bei der Arbeit und beim Autofahren, Reduktion von Motivation, Interesse, Initiative und Energie, Sorgen um den Schlaf sowie die Entwicklung von psychosomatischen Beschwerden (Kopfschmerzen, gastrointestinale Beschwerden) [1]. Die Insomnie darf nicht die Folge einer fehlenden Schlafhygiene sein, d. h. Möglichkeiten und Umstände, einen ausreichenden Schlaf zu haben, sind gegeben. Auszuschließen sind körperliche Ursachen einer Insomnie, andere schlafmedizinische Diagnosen, insbesondere das Restless-Legs-Syndrom und Störungen der circadianen Rhythmik sowie eine Insomnie als Folge von Medikamenten, Drogen oder Substanzen.

Es gibt eine häufige Komorbidität von Insomnie und psychischen Erkrankungen

Die Literatur unterscheidet zwischen einer primären und einer sekundären nichtorganischen Insomnie. Die „Research Diagnostic Criteria“ im Jahr 2004 [3] definierten eine Insomnie als primär, wenn entweder keine psychiatrische Erkrankung vorliegt oder bei Vorhandensein einer solchen der zeitliche Verlauf der Insomnie eine gewisse Unabhängigkeit von dieser zeigt („shows some independence“). Die Insomnie wurde als „sekundär“ bzw. Teil einer psychiatrischen Erkrankung betrachtet, wenn Beginn und Verlauf zeitgleich sind. Diese Unterscheidung wird aber zunehmend kritisiert, meist am Beispiel von Insomnie und Depression. Schlafstörungen sind ein diagnostisches Merkmal der Depression nach DSM 5 und ICD-10. Einige Studien zeigten, dass Schlafstörungen dem Beginn einer Depression zeitlich vorausgehen [4–6] und ein Prädiktor für das Auftreten einer Depression sind [7]. Das Risiko einer nicht-depressiven Person mit Insomnie, in den nächsten Jahren eine Depression zu entwickeln ist 2fach höher als für eine Person ohne Insomnie [8]. Schlafstörungen gehören auch zu den Symptomen, die nach Remission einer depressiven Episode als Residualsymptom oft zurückbleiben [9]. Daher wird der Begriff „sekundäre“ Insomnie vermieden und zunehmend der Begriff „komorbide Insomnie“ verwendet. Der Begriff „primäre Insomnie“ (PI) wird im Folgenden verwendet, wenn die Insomnie die alleinige schlafmedizinische Diagnose ist und die eingehende strukturierte psychiatrische und psychodiagnostische Abklärung keinen Hinweis auf eine psychiatrische Komorbidität ergibt. Der Begriff „komorbide Insomnie“ (KMI) wird bei Vorhandensein einer psychiatrischen Erkrankung verwendet. Voraussetzung für das Stellen der Diagnose KMI ist, dass die Insomnie als eigenständiges Zustandsbild und als eine „Hauptbeschwerde“ betrachtet werden kann.

## Methode

Seit dem 01.10.2020 erhalten konsekutiv alle Patientinnen und Patienten des Schlaflabors des Krankenhauses der Barmherzigen Brüder Wien eine Einladung zur weitergehenden Diagnostik und zur nachfolgenden Teilnahme an einem Therapieprogramm. Die kognitive Verhaltenstherapie der Insomnie wird von der Fakultät für Psychotherapiewissenschaft der Sigmund Freud Privatuniversität gestaltet und durchgeführt (Fachspezifikum Verhaltenstherapie, P. Hulle-Wegl, I. Valeske). Die psychiatrische und psychodiagnostische Abklärung erfolgt ambulant im Krankenhaus der Barherzigen Brüder Wien (S. Deutsch-Lang, M. Kuchling und W. Lang).

Entsprechend internationaler Empfehlungen [10] erfolgt eine mehrschichtige Erfassung der psychiatrischen/psychischen Komorbidität: (1) strukturiertes psychiatrisches Interview unter Verwendung der Internationalen Diagnosen-Checklisten (IDCL) für ICD-10, (2) psychodynamische Diagnostik und (3) Verwendung standardisierter Fragebögen zur Selbstbeurteilung: SCL-90 (Syndrom-Checkliste 90), Beck-Depressions-Inventar (BDI-II) in der für den deutschen Sprachraum angepassten Version, STAI (State Trait Angst Inventar) in der deutschen Version. Verwendet wird der Teil (Trait), der die Angst als Eigenschaft zum Ausdruck bringt. Eingesetzt wird der Whiteley-Index in der deutschsprachigen Version. Er enthält die Unterkategorien Krankheitsangst, Befassung mit den somatischen Symptomen und Krankheitsüberzeugung. Die mehrschichtige Beurteilung erschien uns als wichtig. Die psychiatrische Diagnostik ordnet Kategorien zu, die Testpsychologie ermöglicht – insbesondere auch für die Verlaufsuntersuchung – eine Quantifizierung der Ausprägung psychischer Symptome. Die psychodynamische Diagnostik (S. Deutsch-Lang) soll zum pathogenetischen Verständnis von individuellen Grundkonflikten und Symptomen beitragen.

Erfasst werden dysfunktionale Überzeugungen und Einstellungen zum Schlaf [1, 11; basierend auf 12], die Wahrscheinlichkeit einer Beeinträchtigung des Schlafs durch „stressbelastete“ Situationen (Ford Insomnia Response to Stress Test) [13], die Ausprägung von kognitivem und vegetativem (konditioniertem) „Hyperarousal“ (modifiziert nach Nicassio et al. 1995) [14] und Art bzw. Ausprägung maladaptiver Verhaltensweisen. Verminderte Stressresistenz, konditioniertes „Hyperarousal“, dysfunktionale Überzeugungen und maladaptive Verhaltensweisen sind nach Spielman et al. (1987) die wesentlichen Faktoren für eine „Perpetuierung“ der Insomnie [15].

Die Ausprägung der Schlafstörung wird über ein 14-tägiges Schlaftagebuch, Parameter der Polysomnographie sowie durch zwei standardisierte Fragebögen, Insomnia Severity Index (ISI) und Regensburger Insomnie Skala (RIS; [16]) erfasst. Nichtorganische Insomnie und circadiane Rhythmusstörungen sind – insbesondere im Kontext mit einer psychiatrischen Komorbidität – nicht einfach abgrenzbar. Aus diesem Grund wird auch der Horne-Östberg-Fragebogen eingesetzt, um irreguläre Schlafzeiten erfassen zu können. Die Folgen der Insomnie werden mittels „Fatigue Severity Scale“ und SF-36 (Quality of Life and Function) ermittelt.

Die kognitive Verhaltenstherapie der Insomnie umfasst 9 Doppelstunden über einen Zeitraum von 11 Wochen. Nach der Therapie erfolgen weitere strukturierte Interviews und testpsychologische Untersuchungen. Primärer Endpunkt der Studie ist eine Remission der Insomnie (Insomnia Severity Index, ISI-Score < 8) oder eine Abnahme um mindestens 8 Punkte auf dieser Skala, die von 0–28 die Ausprägung insomnischer Beschwerden beschreibt. Weiterhin untersucht werden Veränderungen der Depressivität (Beck-Depressions-Inventar, BDI-II), der dysfunktionalen Gedanken, des konditionierten „Hyperarousal“ sowie von Fatigue (FSS) und Lebensqualität (SF-36). Eine anonymisierte Datenbank wurde angelegt, um langfristig Hypothesen generieren zu können. Die Zustimmung für dieses Projekt erfolgte über die Ethikkommission des Krankenhauses der Barmherzigen Brüder Wien. Sämtliche datenschutzrechtlichen Bestimmungen sind erfüllt.

## Ergebnisse

Im Zeitintervall (01.10.2021–31.12.2021) erhielten 68 Personen, die im Schlaflabor die alleinige Diagnose „nichtorganische Insomnie“ erhalten hatten, die Einladung zur weiterführenden Diagnostik und Therapie. Der Einladung folgten 43 Personen, alle erhielten die Ausgangsdiagnostik. Insgesamt 23 Personen schlossen die kognitive Verhaltenstherapie der Insomnie bereits ab, 5 Personen brachen die Behandlung ab. Von den restlichen 15 Personen haben 10 die Therapie begonnen, die restlichen 5 sind noch unentschlossen.

Von den 43 Personen sind 26 weiblich. Der Altersmedian ist 40 Jahre (21–72 Jahre). Die Dauer der Schlafstörungen betrug 3–12 Monate (14 %), 1–3 Jahre (9,3 %), mehr als 3 Jahre (41,9 %). Teilweise bestanden die Beeinträchtigungen seit der Kindheit (7 %) oder Jugend (12 %). An häufige Angstträume in der Kindheit erinnerten sich 15 Personen, bei 2 Personen bestand ein Pavor nocturnus bzw. ein Somnambulismus. Es befinden oder befanden sich 34 Patientinnen und Patienten in psychiatrischer Betreuung, 21 haben oder hatten eine psychotherapeutische Behandlung, die nicht speziell auf die Therapie der Insomnie fokussiert war, sondern auf die zugrunde liegende psychiatrische/psychische Störung. Der Median des Summenscores des „Insomnia Severity Index“ betrug 20 (Q1/Q3: 17/23). Die Ausprägung der Schlafstörung ist nach diesem Score als gering (*n* = 6), mäßig-gradig (*n* = 24) oder schwer (*n* = 13) zu klassifizieren.

Das Spektrum der psychischen Komorbidität bei Insomnie ist vielfältig

Das Spektrum der psychiatrischen Komorbidität erwies sich als vielfältig: 9 Personen leiden unter Persönlichkeitsstörungen (Borderline; paranoid, schizoid, emotional instabil oder histrionisch), teilweise in Verbindung mit Depression (*n* = 3), Hypochondrie (*n* = 1) bzw. somatoformer, autonomer Funktionsstörung (*n* = 1). Die häufigste affektive Störung ist die Depression („rezidivierende, depressive Episode“, *n* = 13; erste depressive Episode, *n* = 2), wobei der Schweregrad der Symptomatik zum Zeitpunkt der Untersuchung leicht (*n* = 5), mittelschwer (*n* = 6) bzw. schwer (*n* = 4) war. Die Ergebnisse der strukturierten psychiatrischen Interviews stimmen bei diesen 15 Personen gut mit den Ergebnissen der Selbstbeurteilung im Beck-Depressions-Inventar (BDI-II) überein. Auf Basis des BDI-II ist die Depression bei 5 Personen schwer (BDI-II: Range 29–38), bei 5 mittelschwer (BDI-II: Range 20–28), bei 3 Personen leicht (BDI-II: Range 14–19), bei einer Person minimal (BDI-II: 9–13) und bei einer Person subklinisch (BDI-II: < 9). Ein Patient leidet unter einer bipolaren affektiven Störung. Er ist stabil eingestellt, es bestand zum Zeitpunkt der Untersuchung eine leichte depressive Symptomatik. Weitere affektive Störungen sind Zyklothymie (*n* = 1) und Dysthymie (*n* = 1). Angststörungen sind häufig: soziale Phobie (*n* = 1), generalisierte Angststörung (*n* = 4) sowie Angst und Depression gemischt (*n* = 1). Die Personen, bei denen Ängste im Vordergrund der Psychopathologie stehen, zeigen im STAI (State-Trait-Angst-Inventar) sehr hohe Werte für Angst als Eigenschaft. Des Weiteren finden sich somatoforme (autonome) Funktionsstörungen (*n* = 3), hypochondrische Störung (*n* = 1) und Zwangsstörungen, wobei Zwangsgedanken im Vordergrund standen (*n* = 1). Als Reaktion auf ausgeprägte Veränderungen der Lebensumstände entwickelte sich bei 3 Personen eine Anpassungsstörung mit Angst und Depression. Vier Personen leiden unter einer schweren posttraumatischen Belastungsstörung. Es handelt sich um Frauen, die sexueller und körperlicher Gewalt ausgesetzt waren. Sie wiesen im CAPS (Clinician-Administered PTSD) eine krankheitswertige Ausprägung auf. Bei 9 der 43 Personen besteht keine psychiatrische Erkrankung. Sie leiden unter einer primären Insomnie.

## Diskussion

Vor der SARS-CoV‑2-Pandemie erhielten jährlich ca. 200 Patientinnen und Patienten des Schlaflabors des Krankenhauses der Barmherzigen Brüder die Diagnose „nichtorganische Insomnie“. Das sind ca. 17 % der untersuchten Personen. Die vorgestellte Studie fiel in die Zeit von SARS-CoV‑2 und das Schlaflabor war teilweise gesperrt, die Bettenanzahl reduziert und priorisiert für Patientinnen und Patienten zur Anpassung und Druckeinstellung von Beatmungsgeräten bei Schlafapnoe. Daher hatten in der Zeit vom 01.10.2020 bis 31.12.2021 nur 68 Personen die alleinige Diagnose „nichtorganische Insomnie“ erhalten. Das Spektrum der psychiatrischen Komorbidität ist – wie beschrieben – breit. In Einklang mit der Literatur werden Depression, Dysthymie, saisonale affektive Störungen, generalisierte Angststörung, Panikstörung, bipolare Störungen, Zwangsstörung und posttraumatische Belastungsstörungen und Persönlichkeitsstörungen als häufigste Komorbidität beschrieben [17, 18]. Trotz Vielfalt der Diagnosen werden gemeinsame Mechanismen bei der Entstehung und Chronifizierung der Insomnie diskutiert: chronische Angst, depressives Gedankenkreisen, emotionale Dysregulation (Hemmung von Emotionen, Unfähigkeit, Aggressionen nach außen abzugeben), reduzierte Stressresistenz und in weiterer Folge multimodales konditioniertes „Hyperarousal“ (kognitiv, vegetativ, muskulär) sowie maladaptive Verhaltensweisen [15, 17–19].

Die kognitive Verhaltenstherapie ist Therapie der Wahl bei Insomnie

Alle Personen mit psychiatrischen Erkrankungen befanden oder befinden sich in psychiatrischer Betreuung. Insgesamt 21 der 43 Personen, die sich für eine weiterführende Diagnostik einfanden, befanden oder befinden sich in psychotherapeutischer Behandlung. Eine spezielle Therapie der „nichtorganischen Insomnie“ war den Personen bisher nicht angeboten worden. Ihre Schlafstörung wird mit verschiedensten Medikamenten (Quetialan, Prothipendyl, Pregabalin, Zolpidem, Triazolam, Trazodon, Mirtazapin u. a.) behandelt. Weltweit empfehlen die Schlafmedizinischen Gesellschaften [1, 20–22] die kognitive Verhaltenstherapie der Insomnie als evidenzbasierte First-Line-Therapie der Insomnie und die Pharmakotherapie als Second-Line-Therapie dann, wenn die Psychotherapie nicht wirksam war. Da in Europa aber weiterhin Sedativa als Therapie der ersten Wahl eingesetzt werden, wurde von der „European Sleep Research Society“ eine „European CBT‑I Academy“ (Cognitive Behavioral Therapy of Insomnia) gegründet, um Standards der CBT‑I in Europa einzuführen [23]. In dieser Veröffentlichung (publiziert im Jahr 2020) wird angemerkt, dass in Österreich jährlich nur 40–60 Personen mit der Methode der CBT‑I behandelt werden.

Es stellt sich die Frage, ob die CBT‑I für Personen mit primärer Insomnie und komorbider Insomnie eine vergleichbare Wirksamkeit hat. Die Fallzahl unserer bisherigen Kohorte ist zu gering, um hier eine Antwort geben zu können. Für ein breites Spektrum von Personen mit komorbider Insomnie (KMI) liegen aber bereits Ergebnisse zahlreicher Studien vor, die eine Wirksamkeit zeigen. Die Annahme, dass nach erfolgreicher Behandlung der psychiatrischen Erkrankung auch das Symptom „Insomnie“ remittiert, ist nicht zutreffend. Die Häufigkeit der residualen Insomnie wird mit 50 % bei posttraumatischen Belastungsstörungen [24–26], mit 40 % bei Depression [27] und mit ca. 30 % bei Panikstörung und generalisierter Angststörung [28] angegeben. Die Persistenz der Insomnie ist – wie bei rezidivierenden depressiven Episoden nachgewiesen – ein Prädiktor für ein Rezidiv [29]. Die CBT‑I erwies sich bereits in frühen Studien als wirksam bei der Behandlung der residualen Insomnie [17]. Zwischenzeitlich gibt es zahlreiche, randomisierte Studien, die eine Wirksamkeit der CBT‑I bei Depression [30], posttraumatischen Belastungsstörungen [31], Angststörungen [32, 33], Zwangsstörungen [34] und auch bei Persönlichkeitsstörungen [18] nachweisen.

## Ausblick

Der Erkenntnisgewinn bei der Therapie der nichtorganischen Insomnie basiert in erster Linie auf randomisierten, kontrollierten Studien („evidence to practice“). Diese sind nicht leicht umzusetzen, insbesondere da die bisher etablierte Therapie (kognitive Verhaltenstherapie) nach publizierter Mitteilung [23] nur selten in strukturierter Weise umgesetzt wird. Im vorgestellten Projekt soll der Erkenntnisgewinn über den Ansatz „practice to evidence“ erfolgen. Bei ausreichender Fallzahl sollen auf Basis der Datenbank Hypothesen generiert werden, die langfristig als Grundlage für evidenzbasierte Studien dienen können. Denn trotz bisheriger Evidenz sprechen bisher „nur“ 40–60 % der behandelten Personen auf die Therapie der Insomnie an. Bemerkenswerterweise gibt es wenig Prädiktoren für ein Ansprechen auf die Therapie und kaum Erklärungen für ein Nichtansprechen. In Anbetracht der Häufigkeit der behandlungswürdigen, chronischen nichtorganischen Insomnie in der Bevölkerung von 3 % [35] und einer zunehmend „schlaflosen“ Gesellschaft hat das Thema Bedeutung. Motivation für die aktuelle Übersicht und Vorstellung des Projekts ist auch, das Interesse an Kooperationen zu wecken.

## Fazit für die Praxis

- Wie vermutlich in anderen Schlaflabors in Österreich gibt es einen Anteil von Personen, die bei Entlassung die Diagnose „nichtorganische Insomnie“ (F51.0) erhalten. Alle Personen haben als eine Hauptbeschwerde chronische Ein‑, Durchschlafstörungen bzw. ein frühzeitiges Erwachen mit Tagesmüdigkeit und reduzierter Lebensqualität.
- Sehr häufig besteht eine psychiatrische Erkrankung, am häufigsten Depression, Angst, posttraumatische Belastungsstörungen und Persönlichkeitsstörungen.
- Die ausschließliche Behandlung der psychiatrischen Erkrankung ist oft nicht ausreichend, um auch das Problem der residualen Insomnie zu beseitigen.
- Trotz Vielfalt der Diagnosen werden gemeinsame Mechanismen bei der Entstehung und Chronifizierung der Insomnie diskutiert: chronische Angst, depressives Gedankenkreisen, emotionale Dysregulation, reduzierte Stressresistenz und in weiterer Folge ein konditioniertes „Hyperarousal“ (kognitiv, vegetativ, muskulär) und maladaptive Verhaltensweisen.
- Die kognitive Verhaltenstherapie bietet eine Möglichkeit, die insomnischen Beschwerden gezielt zu behandeln. Trotz guter Wirksamkeit bleiben die Prädiktoren für ein Ansprechen/ein Nichtansprechen auf diese Therapie unklar. Es gibt somit eine Notwendigkeit für weitere Entwicklungen, die in Anbetracht der Bedeutung des Themas für die Betroffenen und die Gesellschaft erforderlich sind.
